# Study of the Cardiotoxicity of Venenum Bufonis in Rats using an 1H NMR-Based Metabolomics Approach

**DOI:** 10.1371/journal.pone.0119515

**Published:** 2015-03-17

**Authors:** Ge Dong, Dandan Wei, Junsong Wang, Pingping Guo, Minghui Li, Minghua Yang, Lingyi Kong

**Affiliations:** 1 State Key Laboratory of Natural Medicines, Department of Natural Medicinal Chemistry, China Pharmaceutical University, Nanjing, Jiangsu, P.R. China; 2 Center for Molecular Metabolism, School of Environmental and Biological Engineering, Nanjing University of Science and Technology, Nanjing, Jiangsu, P.R. China; National Research Council of Italy, ITALY

## Abstract

Venenum Bufonis, a well-known traditional Chinese medicine, has been widely used in Asia and has gained popularity in Western countries over the last decade. Venenum Bufonis has obvious side effects that have been observed in clinical settings, but few studies have reported on its cardiotoxicity. In this work, the cardiotoxicity of Venenum Bufonis was investigated using a ^11^H NMR-based metabolomics approach. The ^1^H NMR profiles of the serum, myocardial extracts and liver extracts of specific-pathogen-free rats showed that Venenum Bufonis produced significant metabolic perturbations dose-dependently with a distinct time effect, peaking at 2 hr after dosing and attenuating gradually. Clinical chemistry, electrocardiographic recordings, and histopathological evaluation provided additional evidence of Venenum Bufonis-induced cardiac damage that complemented and supported the metabolomics findings. The combined results demonstrated that oxidative stress, mitochondrial dysfunction, and energy metabolism perturbations were associated with the cardiac damage that results from Venenum Bufonis.

## Introduction

Venenum Bufonis (VB, Chinese name ‘‘Chan Su”), the dried secretions of the auricular and skin glands of *Bufo gargarizans* Cantor or *Bufo melanostrictus* Schneider, is a well-known traditional Chinese medicine (TCM) that has been widely used in clinic as a cardiotonic, diuretic, anodyne, and antineoplastic agent [1–3]. In addition to its popularity in China, Japan, and other Asian countries, VB has also become increasingly used in the United States and other Western countries over the last decade [4]. Unfortunately, VB has demonstrated side effects in clinical settings resulting from its toxicity; including nausea, vomiting, diarrhea, abdominal discomfort, and general paralysis. The major complaints of patients who have taken VB are its cardiac effects, which are similar to those of digitalis, exhibiting bradycardia, atrioventricular conduction blockage, ventricular tachycardia, and even leading to sudden death [5,6].

Many chemical components, including cardiotonic steroids (bufosteroids), indoleamines, peptides, amino acids, fatty acids, polysaccharides, and sterols, were found in VB [7–9]. Among them, bufosteroids, including bufalin, cinobufotalin, resibufogenin and cinobufagin [10], are the main therapeutic and toxic components of VB. Functioned as Na^+^/K^+^-ATPase inhibitors, bufadienolides, can trigger Na^+^/Ca^2+^ exchange in cardiac myocytes and thus facilitate the inflow of calcium ions, resulting in an increase in the level of intracellular calcium ions [11]. However, the mechanism underlying the cardiac toxicity of VB remains unclear due to the complex composition in it. In addition, these individual compounds alone cannot explain the mechanism of VB as a whole. To elucidate the mechanism of cardiac damage induced by VB and discover potential biomarkers, a proton nuclear magnetic resonance spectroscopy (^1^H-NMR)-based metabolomics approach was utilized to study the metabolic changes that occur in serum, heart and liver of rats subjected to varying doses of VB.

Metabolomics provides a whole-organism biological description of multivariate metabolic responses to a perturbation via analytical techniques such as NMR, LC-MS, and GC-MS. By monitoring a variety of metabolites that can be related to toxicity or other perturbations, metabolomics has been successfully used for the discovery of biomarkers and in preclinical settings, particularly for the evaluation of toxicity, safety, and efficacy [12–14].

The application of metabolomics to the toxicological study of TCM has obvious benefits over traditional technologies. Metabolomics can globally evaluate the biological effects gleaned from metabolic profiles that contain massive amounts of biological information, thus simplifying the mechanistic study of complex TCM. The ability of metabolomics to dynamically monitor metabolic events in response to the administration of a drug also makes it suitable for toxicological studies involving the effects of time. As a result, metabolomics approaches have been successfully applied to studying the toxicities of TCMs such as cinnabar [15], Hei-Shun-Pian [16], *Aconite Lateralis* [17], *Aconitum carmichaelii Debx*, [18] and *Pinellia ternate* [19].

^1^H NMR spectroscopy has proven to be a powerful and popular technique in metabolomics studies. ^1^H NMR provides unique structural information regarding the metabolites and is a rapid, non-destructive, high-throughput method that requires minimal sample preparation [13,20]. To better delineate the onset and progress of myocardium damage induced by VB, a ^1^H NMR-based metabolomics approach was used in this study. NMR profiling of serum [21], myocardial extracts and liver extracts in combination with orthogonal projection latent structure analysis (OPLS-DA) revealed that oxidative stress, mitochondrial dysfunction, energy shortages in myocardial cells were associated with the cardiac toxicity of VB.

## Materials and Methods

### Chemicals and reagents

The crude drug form of VB was purchased from Jiangsu Medicine Company (Nanjing, China) and authenticated by Prof. Ming-Jian Qin (Department of Medicinal Plants, China Pharmaceutical University). 3-Trimethylsilylpropionic acid (TSP) was obtained from Sigma (St. Louis, MO, USA). Deuterium oxide (D_2_O, 99.9%) was purchased from Sea Sky Bio Technology Co. Ltd (Beijing, China). Pentobarbital sodium was obtained from Fuoshan Chemical Factory (Fuoshan, China). Ultra-pure distilled water, prepared using a Milli-Q purification system, was utilized in the experiments.

### Ethics Statement

All animal care and use was conducted under a license granted by Jiangsu Science and Technology Office (China) with approval by the Animal Ethics Committee of China Pharmaceutical University (Permit number: SYXK2007–0025). All experiments were conducted in compliance with the National Institutes of Health (NIH) guidelines for the Care and Use of Laboratory Animals. All surgery was performed under sodium pentobarbital anesthesia, and all efforts were made to minimize suffering.

### Animals

Ten-week-old male specific-pathogen-free (SPF) rats weighing 240–300 g were obtained from the Experimental Animal Center of Yangzhou University (Yangzhou, China). They were given free access to standard diet and water and were housed in a room (5 rats to one cage) with controlled humidity (50 ± 5%) and temperature (23 ± 2°C) under a 12/12-h light/dark cycle. The animals were allowed to acclimate for 10 days prior to dosing.

### Preliminary experiments

To optimize the experimental conditions, we performed preliminary experiments in strict accordance with the 3R principle (Reduction, Replacement, Refinement). A few of rats taken high dose of VB were dead in the preliminary experiments. The final reason of death is the digitalis toxicity-like cardiac effects and the central nerve system toxicity caused by VB. We found a close association between the death occurrence and the body weight of rats: the heavier a rat, the more intolerant the rat to the toxicity of VB. The rats of heavy weight exhibit serious symptoms of toxicity: urinary incontinence, limb twitch, spasticity, slobber, even death; the rats of light weight only exhibit mild symptoms of toxicity: sluggish, shortness of breath, limb twitch.

According to the results of preliminary experiment, we chose the ten-week-old male SPF rats weighing 240–300 g to do the formal experiment aiming to minimize the death occurrence in dosed rats. As a result, rats taken high doses of VB exhibited sluggish, shortness of breath, and slightly limb twitches in the formal experiment without death within 2 hr after the administration of VB.

### Drug administration and sample collection

Rats were treated with or without VB (NC group, n = 10), at random, after acclimatization. The drug was dissolved in ultra-pure distilled water and intragastrically administered to rats at doses of 100, 300, and 500 mg/kg (LT, MT, and HT for the low, medium, and high doses, respectively) at equal volume (10 ml/kg bodyweight). Normal control rats were administered equivalent volumes of distilled water. The rats were prevented from eating for 12 hr before the experiments, but they were allowed free access to water. In the formal experiment, the animals were continuously monitored and their behaviors were continuously observed.

At the end of each timed experiment, the rats were deeply anesthetized with pentobarbital sodium (30 mg/kg, i.p.) and then sacrificed in groups, after which blood, myocardial and liver samples were collected. Samples from the low-dose rats were collected at the 2^nd^ (LT-2, n = 10), 6^th^ (LT-6, n = 10), and 12^th^ hours after dosing (LT-12, n = 10); and at the 2^nd^ hour, samples from three dosed rats were collected (LT-2, MT-2, HT-2, n = 10 in each group). Blood was collected from the abdominal aorta, and serum samples were obtained by centrifugation (12,000 rpm, 10 min, 4°C) and stored at −80°C before analysis. Heart and liver tissues were quickly removed, weighed, and rinsed with cold phosphate-buffered saline (PBS). Left ventricle of heart samples and left lobe of liver samples were frozen and stored at −80°C for ^1^H NMR studies. The right heart ventricle and right liver lobe of two rats from each group were used for histological examination, fixed in neutral buffered formalin (10% formalin in 0.08 M sodium phosphate, pH 7.4). Right heart ventricle of other rats of each group were stored at −80°C before the measurements of reactive oxygen species (ROS), malondialdehyde (MDA).

At the second hour after the administration of distilled water (NC) or three dosages of VB (LT-2, MT-2, and HT-2), four rats from each group were randomly selected and anesthetized with pentobarbital sodium (30 mg/kg, i.p.). Needle electrodes were inserted under the skin in the lead II position. Heart rate and electrocardiograph (ECG) recordings were obtained using a BL-420S Biological Function Experiment system (Technology & Market Co., Ltd., Chengdu, China). The same experiments were also performed on rats one day before dosing, as a control.

### Clinical biochemistry and histopathology

The levels of serum γ-glutamyl transferase (γ-GT), creatine kinase (CK), and non-esterified fatty acid (NEFA, FFA) were measured using commercially available kits (Nanjing Jianchen Biotech Inc., China). MDA, ROS and protein concentrations in cardiac myocytes were measured using Lipid Peroxidation MDA Assay Kit, Reactive Oxygen Species Assay Kit and Enhanced BCA Protein Assay Kit (Beyotime Institute of Biotechnology, Haimen, China).

Heart and liver tissues were immersed in 10% neutral buffered formaldehyde for 48 h, embedded in paraffin, and sliced at a thickness of 5 μm. The sliced sections were stained with hematoxylin and eosin (HE) and examined by light microscopy (× 200 magnification, Olympus DX45). The histopathology results were evaluated by Professor Ning Su (Southeast University, Nanjing, China) who was blinded.

### ^1^H NMR spectroscopy of serum samples

After thawing, serum samples (300 μl) were added to 150 μl of buffer solution (0.2 mol/l Na_2_HPO_4_ and 0.2 mol/l NaH_2_PO_4_, pH 7.4) and 150 μl of TSP (3-trimethylsilylpropionic acid, 1 mg/ml, Sigma-Aldrich) in D_2_O. After vortexing, the mixture was allowed to stand for 20 min and before being centrifuged at 12,000 rpm for 10 min at 4°C to remove any precipitate. Aliquots of 550 μl of the supernatant were placed into 5-mm NMR tubes.

All ^1^H NMR spectra were recorded at 25°C on a Bruker AV 500 MHz spectrometer. A water-suppressed Carr-Purcell-Meibom-Gill (CPMG) spin-echo pulse sequence (90(τ-180-τ)n-acquisition) with a total spin-echo delay (2 nτ) of 10 ms was used to suppress broad signals from macro molecules (i.e., proteins or lipoproteins), whereupon the signals of micro molecules were clearly observed. ^1^H NMR spectra were measured with 128 scans producing 32,000 data points over a spectral width of 7,500 Hz [22]. The spectra were Fourier transformed after multiplication by an exponential window function with a line broadening of 0.5 Hz and were then manually phased and baseline corrected using TOPSPIN software (version 3.0, Bruker Biospin, Germany).

### ^1^H NMR spectroscopy of myocardial extracts and liver extracts

Preweighed heart tissue (200 mg) was homogenized in 50% acetonitrile/50% D_2_O (1.5 ml) and centrifuged at 12,000 rpm for 10 min at 4°C. The supernatant was collected, lyophilized and reconstituted in D_2_O (600 μl) containing TSP (1 mg/ml) as an internal standard and buffer (0.2 mol/l Na_2_HPO_4_ and 0.2 mol/l NaH_2_PO_4_, pH 7.4). All mixed samples were vortexed and centrifuged at 12,000 rpm for 10 min at 4°C. The collected supernatants (550 μl) were transferred into 5-mm NMR tubes. The liver tissue used the same method with the myocardial tissue.

^1^H NMR analysis of the myocardial extracts and liver extracts were performed on a Bruker Avance spectrometer operating at 500 MHz. All spectra were acquired with 64 scans at 25°C using a 1D NOESYPRESAT pulse sequence with water suppression (Bruker’s pulse program noesypr1d), a relaxation delay of 2.0 s, and a mixing time of 100 ms. Free induction decays (FIDs) were collected with 64,000 data points, an acquisition time of 1.63 s, and a spectral width of 10,000.0 Hz [22,23]. Prior to Fourier transformation, an exponential line-broadening function of 0.3 Hz was applied to the FID. All ^1^H NMR spectra were manually phased and baseline-corrected using TOPSPIN software (version 3.0, Bruker Biospin, Germany).

### Data processing and analysis

The processing methods used on the raw NMR data were based on protocols described in our previous work [24]. Briefly, all ^1^H NMR spectra were manually phase baseline corrected, referenced to TSP (^1^H, δ 0.0) using Bruker Topspin 3.0 software (Bruker GmbH, Karlsruhe, Germany), automatically exported to ASCII files using MestReNova (Version 8.0.1, Mestrelab Research SL), and then imported into “R” (http://cran.r-project.org/) before being aligned further with an R script developed in-house. The spectra were then binned into 0.005 ppm integrated spectral buckets between 0.2 and 10 ppm. Regions of residual water resonance (between 4.35 to 5.70 ppm in myocardial extracts spectra, 4.16–5.70 ppm in liver extracts spectra, and 4.19–9.70 in serum spectra) were removed.

The data matrix consisting of all spectra were mean centered and pareto-scaled to simplify the meaning and interpretation of the modeling coefficients and to reduce multicollinearity. The integral values of each spectrum were then probability quotient normalized to account for different sample dilutions.

Multivariate statistical data analysis, OPLS-DA method, was performed using “R” package “muma” [25]. OPLS-DA method could remove systematic variations unrelated to interested status through an orthogonal filter and identified the most significant variations between the treatment groups. To check the validity of the model and avoid overfitting the PLS model, an assessment of the 7-fold cross-validated scores from the model was used. The validity of the model against overfitting was assessed by the parameter *R* ^*2*^ *Y*, and the predictive ability of the model was described by *Q* ^*2*^ *Y*. Permutation testing was based on the comparison of the predictive capabilities of an OPLS-DA model using real class assignments with models calculated after random permutation of the class labels.

A parametric Student's t-test or a nonparametric Mann-Whitney test (dependent on conformity to the normal distribution) was performed on the signal integrals to evaluate the differences in metabolites between groups. The integration areas of the detected metabolites with marked differentiating ability were first tested for the normality of their distribution. If their distribution followed the normality assumption, a parametric Student's t-test was applied; otherwise, a nonparametric Mann-Whitney test was performed to detect statistically significant increases or decreases in metabolites between groups.

## Results

### ECG and heart rate parameters

**Fig 1:**
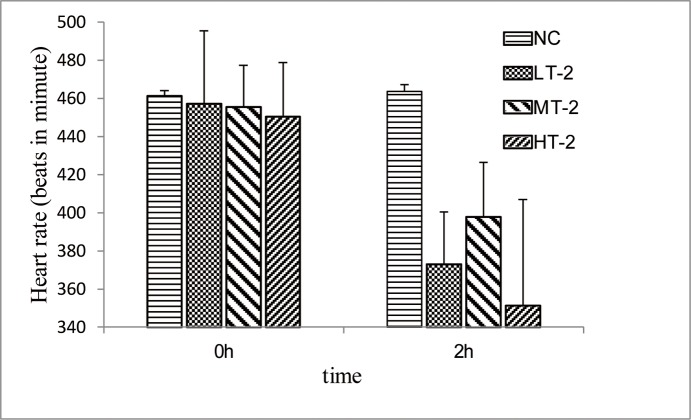
Change in heart rates of rats 2 hr after administration of VB. After being administered equivalent volumes of distilled water, rats in the NC group did not exhibit any changes in heart rate. After being administered VB, rats in groups LT-2, MT-2, and HT-2 exhibited significant decreases in heart rate (*p*<0.01).

**Fig 2:**
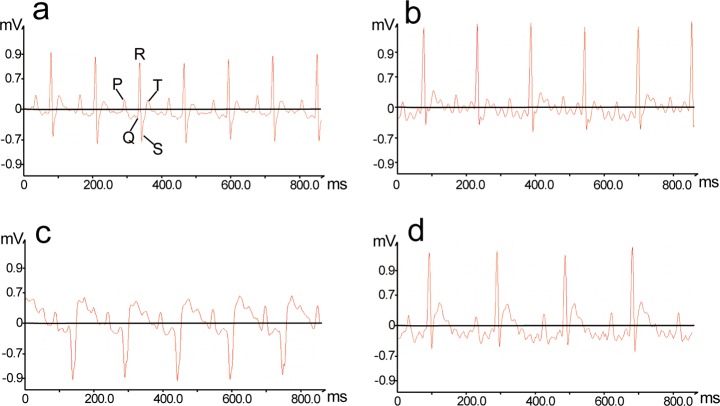
Representative electrocardiogram tracings 2 hr after administration of VB. (a) NC group, (b) LT-2, (c) MT-2, (d) HT-2. After being administered VB, rats in groups LT-2, MT-2, and HT-2 exhibited marked disturbances in their ECG pattern (e.g., ST-segment elevation and pathological Q waves).

An ECG trace records the electrical activity of the heart. Rats in the NC group did not exhibit any changes in heart rate (Fig. 1) or ECG pattern (Fig. 2), while those taken from the VB group exhibited marked disturbances in their ECG patterns (e.g., ST-segment elevation and pathological Q waves)[26] and significant decreases in heart rate.

### Cardiac and liver histopathological evaluation

**Fig 3:**
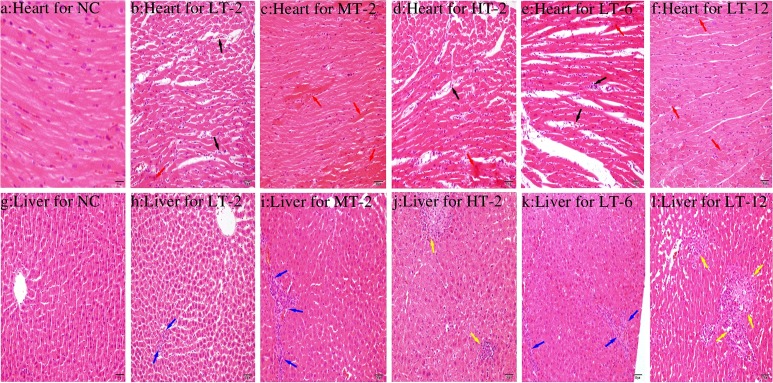
Photomicrographs of sections of myocardium (a-f) and liver (g-l) taken at 200 x magnification. (a) Normal myocardium cytoarchitecture; (b-f) Pathological changes in the myocardium after the administration of VB in different groups; (g) Normal liver cytoarchitecture; (h-l) Pathological changes in the liver after the administration of VB in different groups. VB treated heart showed obviously dilated intercellular spaces (black arrow), abundant eosinophilic cytoplasm and marked inflammatory cell infiltrations (red arrow). Live showed moderate piecemeal necrosis (yellow arrow) and slight inflammatory cell infiltrations in the portal tracts (black arrow).

Histopathological examinations on heart and liver were blindly assessed by two independent experts to reveal tissue specific pathological changes induced by VB. Compared with the NC group (Fig. 3a), obviously dilated intercellular spaces were observed in severely damaged myocardium of the VB-treated rats (Fig. 3b, d and e) accompanied by abundant eosinophilic cytoplasm and marked inflammatory cell infiltrations (Fig. 3b-f). Moderate piecemeal necrosis (Fig. 3j and l) and slight inflammatory cell infiltrations in the portal tracts of livers (Fig. 3h, i and k) were shown in VB-treated rats as compared with NC rats (Fig. 3g). However, neither dose-dependence nor time-dependence was observed for VB-induced histopathological injury.

### Biochemical analysis

The observed changes in biochemical parameters are shown in Table 1. Significantly increased levels of serum CK, γ-GT, FFA, MDA and ROS were found in groups given VB compared with the NC group. As a reliable indicator of myocardial injury, the serum CK level peaked at 6 hr after the administration of VB, consistent with previous reports [27,28]. The liver toxicity of VB was demonstrated by the presence of significantly elevated γ-GT levels, complemented by pathological evaluation of the liver. The increased FFA levels observed in serum indicated that a disturbance in fatty acid metabolism was induced by VB. The final levels of ROS in myocardial tissue were expressed as fluorescence/mg of protein: ROS was firstly determined using 2, 7-dichlorofluorescin diacetate (DCFH-DA) [29] and protein concentrations were determined by bicinchoninic acid (BCA) assay. The levels of ROS in heart of VB treated rats were significantly increased compared with the NC group, particularly in the high dosage group. In the meanwhile, MDA, an end product of lipid peroxidation, presented significantly increased levels in low dosage VB treated rats sacrificed at 6h and 12h compared to NC group. The elevated ROS and MDA levels demonstrated the oxidative stress induced by VB treatment.

**Table 1:** Biochemical parameters measured in NC and VB treated groups.

	NC	LT-2	LT-6	LT-12	MT-2	HT-2
**CK (U/ml)**	0.464±0.293	0.372±0.287	0.852±0.182**	0.564±0.135	0.399±0.200	0.181±0.093*
**FFA (μmol/L)**	831±74.3	1012±62.1***	894±68.6	943±166.3	1102±173.3**	849±94.9
**γ-GT (U/L)**	15.0±7.28	22.0±0.868*	32.6±2.80***	33.9±1.75***	38.5±1.87***	39.9±2.96***
**MDA (nmol/mg)**	7.59±0.016	7.46±0.017	8.39±0.097***	8.81±0.15***	7.29±0.10	7.41±0.070
**ROS**	23073.155	35126.131	33145.213	34125.189	33538.191	51057.101

Data are presented as the mean ± SD with seven animals per group. ROS values expressed as the level of fluorescence/mg protein.

(*) *p* <0.05,

(**) *p* <0.01,

(***) *p* <0.001 compared to the NC group.

### Metabolites identification

**Fig 4:**
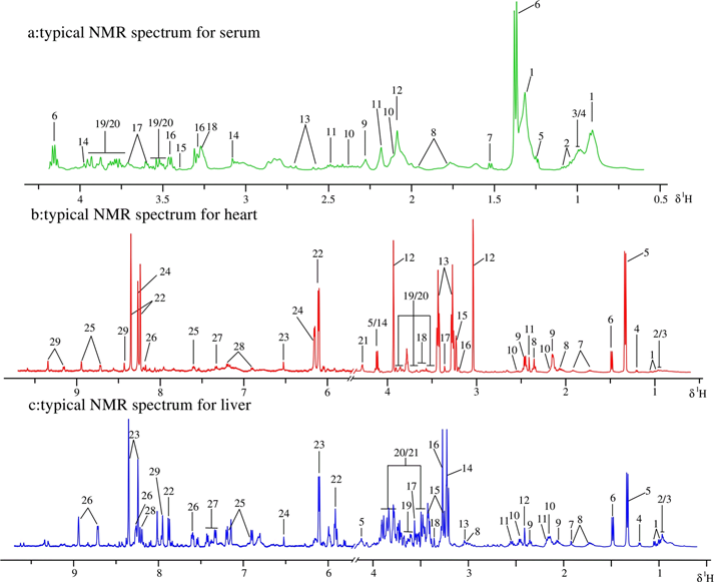
Typical 500 MHz ^1^H NMR spectra of serum samples (a), myocardial extract samples (b) and liver extract samples (c) obtained from all groups. Metabolites were respectively listed in S1–S3 Tables along with their chemical shifts.

**Fig 5:**
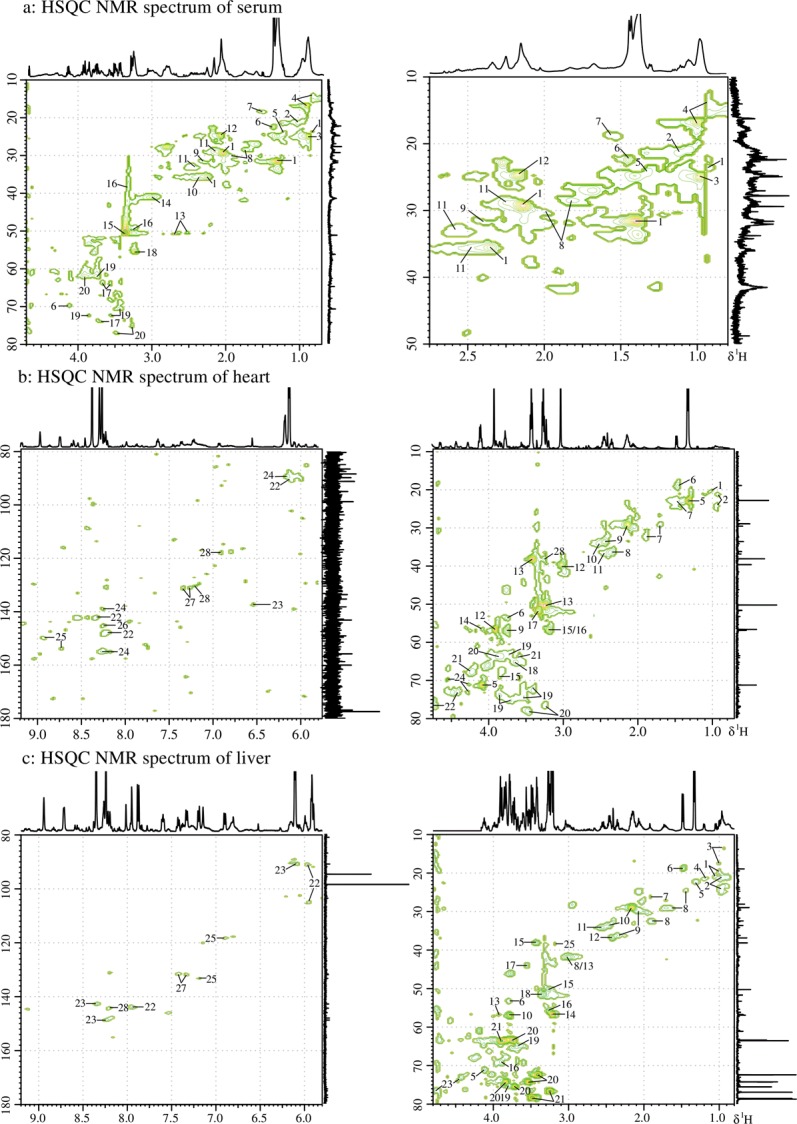
HSQC NMR spectra of serum (a), heart (b), liver (c) at 500 MHz showing the assignment of important metabolites, which were listed in S1–S3 Tables.

The ^1^H NMR spectra of the analyzed serum, myocardial extracts and liver extracts were highly complex, dominated by numerous signals originating primarily from micro molecules, as depicted in Fig. 4. Resonances were assigned by querying publicly accessible metabolomics databases such as Human Metabolome Database (HMDB, http://www.hmdb.ca), aided by Chenomx NMR suite 7.5 (Chenomx Inc., Edmonton, Canada), confirmed by the two-dimensional NMR techniques TOCSY (total correlation spectroscopy) and HSQC (heteronuclear single quantum correlation) (Fig. 5), and supported by the previous literature [30–32]. The assignments and integrations of peaks were assisted through the use of the STOCSY (statistical total correlation spectroscopy) technique (S1 Fig.). 20 metabolites in the serum, 29 metabolites in the myocardial extracts and 29 metabolites in the liver extracts were assigned and are listed in S1–S3 Tables along with their chemical shifts. Assigned metabolites in serum: (1), low-density-lipoproteins/ very-low-density lipoproteins (LDL/VLDL); (2), Valine (Val); (3), Leucine (Leu); (4), Isoleucine (Ile); (5), 3-Hydroxybutyrate (3-HB); (6), Lactate (Lac); (7), Alanine (Ala); (8), Arginine (Arg); (9), Acetone (DMK); (10), Glutamate (Glu); (11), Glutamine (Gln); (12), N-Acetyl Glycoproteins (NAGP); (13), Citrate (Cit); (14), Creatine (Cr); (15), Methanol (MeOH); (16), Taurine (Tau); (17), Glycerol (Gle); (18), Betaine (Bet); (19), α-Glucose (Glc); (20), β-Glucose. Assigned metabolites in myocardial extracts: (1), Val; (2), Leu; (3), Ile; (4), 3-HB; (5), Lac; (6), Ala; (7), Lysine (Lys); (8), Glu; (9), Gln; (10), Glutathione (GSH); (11), Succinate (Suc); (12), Cr; (13), Tau; (14), Cystine; (15), Bet; (16), Choline (Cho); (17), MeOH; (18), Gle; (19), α-Glucose; (20), β-Glucose; (21), Threonine; (22), Inosine (Ino); (23), Fumarate (Fum); (24), Cyclic AMP (cAMP); (25), Niacinamide (Nia); (26), Hypoxanthine (Hyp); (27), Phenylalanine (Phe); (28), Tyrosine (Tyr); (29), NAD^+^. Assigned metabolites in liver extracts: (1), Val; (2), Leu; (3), Ile; (4), 3-HB; (5), Lac; (6), Ala; (7), Acetate (Ace); (8), Lys; (9), Glu; (10), Gln; (11), GSH; (12), Suc; (13),Cr; (14), Cho; (15), Tau; (16), Bet; (17), Glycine (Gly); (18), MeOH; (19), Gle; (20), α-Glucose; (21), β-Glucose; (22), Uridine (Uri); (23), Ino; (24), Fum; (25), Tyr; (26), Nia; (27), Phe; (28), Hyp; (29), Xanthine (Xan).

### Multivariate analysis of ^1^H NMR data of all groups

**Fig 6:**
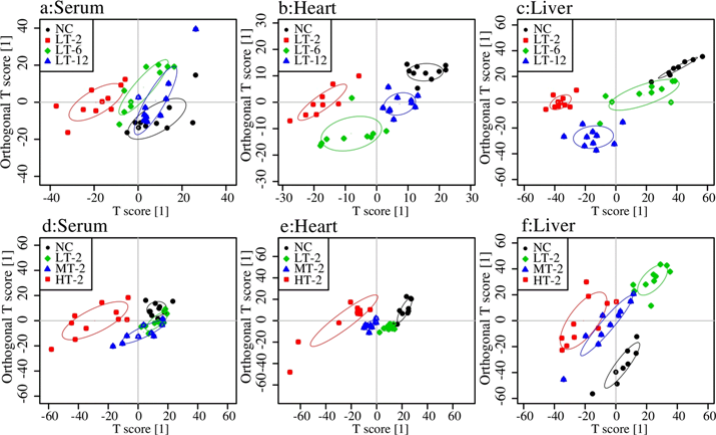
Score plots for OPLS-DA analysis of ^1^H NMR data for serum, myocardial extracts and liver extracts of NC and VB treated groups. Two independent analyses were performed to study the time (NC, LT-2, LT-6 and LT-12) (a, b and c) and dose (NC, LT-2, MT-2 and HT-2) (d, e and f) effects of VB-induced toxicity. (a, d): score plots for serum; (b, e): score plots for myocardial extracts; (c, f): score plots for liver extracts. Ellipses around sample points stood for the 75% confidence.

The ^1^H NMR data from the normal control (NC) rats and the low dose VB-administered rats were evaluated at three time points (LT-2, LT-6, LT-12) using OPLS-DA analysis to dynamically investigate the time-dependent toxicity of VB at a dosage comparable to that used in clinic. In the score plots, each point represents a sample and each clustering of a group of samples represents a metabolic pattern corresponding to each group. In the OPLS-DA score plots (Fig. 6a and b) of serum and myocardial extracts, the LT-2 group was the furthest apart from the NC group, with LT-6 and LT-12 groups in between and the LT-12 group the closest to the NC group. VB produced maximal metabolic disturbance at 2 hr after dosing, which was then gradually moved towards the normal in serum and myocardial extracts. This trend for metabolic alterations was in consistent with that for the behavioral changes observed in the preliminary experiments: the most violent at 0–2 hr and then attenuated gradually. In the score plots for liver extracts (Fig. 6c): LT-2 is also the furthest away from NC with LT-6 and LT-12 groups in between but the LT-6 group the closest to the NC group.

^1^H NMR data of serum, myocardial and liver extracts of NC, LT-2, MT-2 and HT-2 groups were analyzed together to detect the dose-effect relationship of VB-induced toxicity. The score plots for the serum (Fig. 6d), myocardial extracts (Fig. 6e) and liver extracts (Fig. 6f) showed a clear separation of VB treated groups from NC, suggesting a severe metabolic perturbation induced by VB. The HT-2 group was the furthest away from that of the NC group, with LT-2 closest to NC group and MT-12 group in the middle of the HT-2 and NC groups, which showcased a dose-dependent toxicity induced by VB. This dose effect was consistent with behavioral observations of the three dosed groups: rats administered low doses of VB showed no noticeable behavioral abnormalities, those given medium doses were sluggish, and those given high doses exhibited limb twitches and, in some cases, death.

### Metabolic changes in VB groups

The OPLS-DA analysis above investigated the time and dose effects of VB induced toxicity. To further explore the metabolic events happened in a given dose of VB at a specific time period, the NMR data of these VB groups were compared with that of NC by OPLS-DA analysis, individually.

#### Metabolic changes in serum of VB treated rats

**Fig 7:**
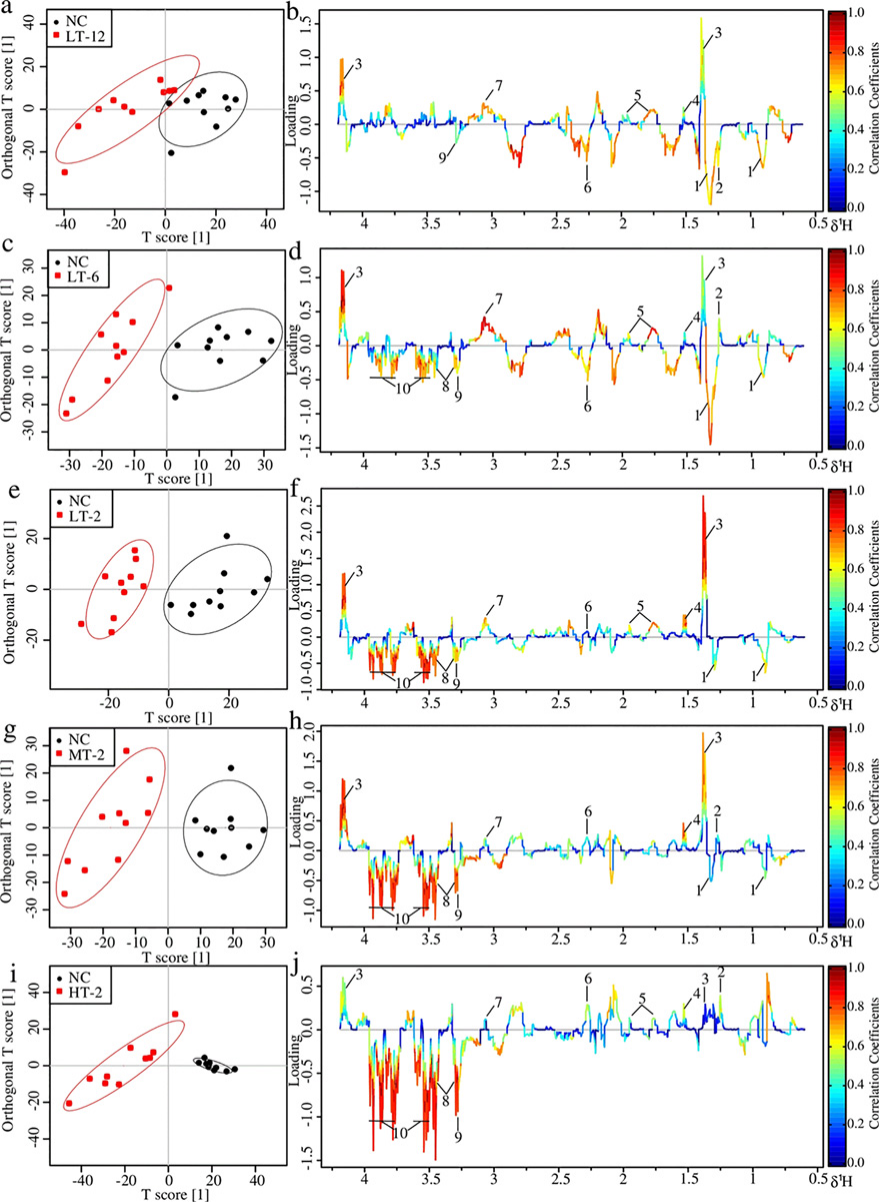
OPLS-DA analysis of serum ^1^H NMR data of LT-12, LT-6, LT-2, MT-2 and HT-2 groups in couple with NC group: score plots (a, c, e, g and i) and loading plots (b, d, f, h and j). In score plots, ellipses around sample points stood for the 90% confidence. Metabolite variation was visualized by the loading plots, which are color-coded according to the absolute value of the correlation coefficient; a reddish signal indicates a more significant contribution to the class separation than a bluish signal. Metabolites: 1, LDL/VLDL; 2, 3-HB 3, Lactate; 4, Alanine; 5, Arginine; 6, Acetone; 7, Creatine; 8, Taurine; 9, Betaine; 10, Glucose.

**Fig 8:**
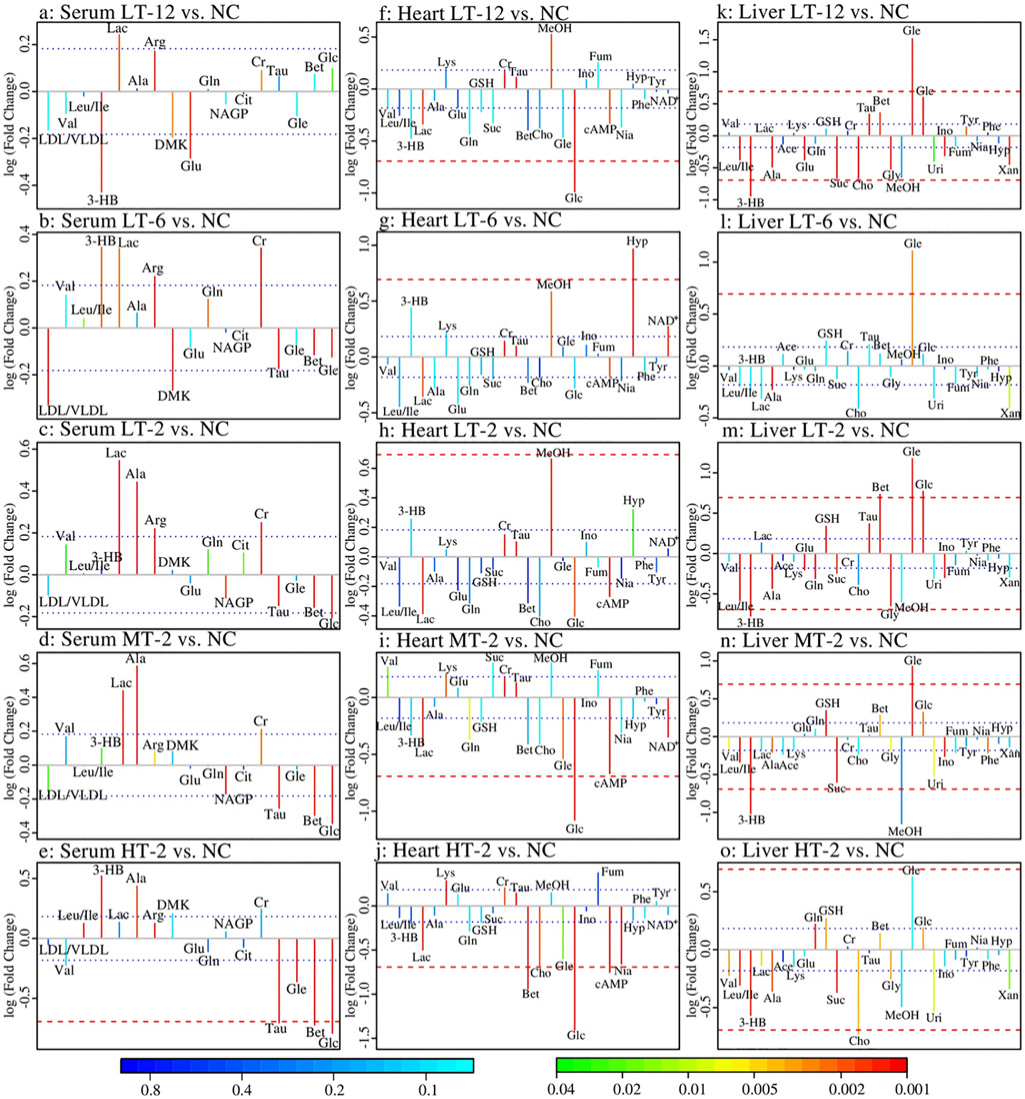
Fold change plots color-coded with *p*-values adjusted by Benjamini-Hochberg method. Fold change plots indicating significance of altered metabolites in serum (a-e), myocardial extracts (f-j) and liver extracts (k-o) of NC rats vs. LT-12 rats (a, f and k), NC rats vs. LT-6 rats (b, g and l), NC rats vs. LT-2 rats (c, h and m), NC rats vs. MT-2 rats (d, i and n) and NC rats vs. HT-2 rats (e, j and o) after VB treatment. The blue and red dashed lines represented variations of 20% and 100%, respectively. Metabolites abbreviation: 3-HB: 3-Hydroxybutyrate; Ace: acetate; Ala: alanine; Arg: arginine; Bet: betaine; Cr: creatine; Cho: choline; Cit: citrate; DMK: acetone; Fum: fumarate; Glc: glucose; Gle: glycerol; Gln: glutamine; Glu: glutamate; Gly: glycine; GSH: glutathione; Hyp: hypoxanthine; Ino: inosine; Lac: Lactate; Lys: lysine; LDL/VLDL: low-density-lipoproteins/ very-low-density lipoproteins; Leu/Ile: Leucine/Isoleucine; MeOH: methanol; NAGP: N-Acetyl Glycoproteins; Nia: niacinamide; Phe: phenylalanine; Suc: succinate; Tyr: tyrosine; Tau: taurine; Val: valine; Uri: Uridine; Xan: xanthine.

NC and each VB administrated group showed well separation in the score plots of OPLS-DA analysis of serum NMR data (Fig. 7). Metabolite variation contributed to the separation of each two groups were visualized by the loading plots (Fig. 7b, d, f, h and j), color-coded according to the absolute value of correlation coefficients where a hot-colored signal (red) indicates more significant contribution to the class separation than a cold-colored one (blue). The fold change values of metabolites in VB groups relative to the NC group and the associated *p*-values adjusted by Benjamini-Hochberg were calculated and visualized by color table (S2 Fig.) and fold change plots (Fig. 8), where each compound corresponded to a line segment color coded from 0.001 in red to high values (0.1–0.8) in blue, according to the *p*-values.

The upper section of the color-coded loading plots and fold change plots represented metabolites decreased in serum of the VB treated groups: 3-HB, lactate, alanine, arginine and creatine; whereas the low section represented metabolites increased: acetone, betaine, taurine and glucose.

#### Metabolic changes in heart of VB treated rats

**Fig 9:**
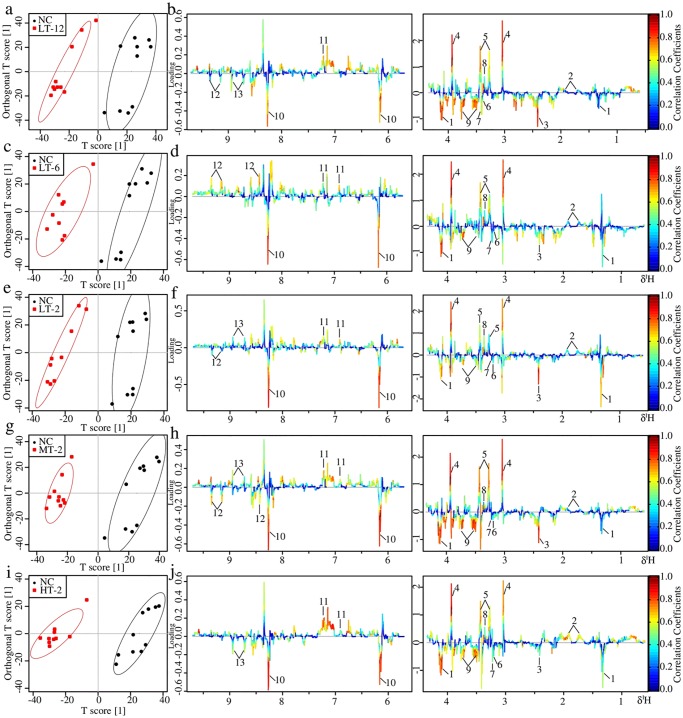
OPLS-DA analysis of myocardial extracts ^1^H NMR data of LT-12, LT-6, LT-2, MT-2 and HT-2 groups in couple with NC group: score plots (a, c, e, g and i) and loading plots (b, d, f, h and j). In score plots, ellipses around sample points stood for the 90% confidence. Metabolite variation was visualized by the loading plots, which are color-coded according to the absolute value of the correlation coefficient; a reddish signal indicates a more significant contribution to the class separation than a bluish signal. Metabolites:1, Lactate; 2, Lysine; 3, Succinate; 4, Creatine; 5, Taurine; 6, Choline; 7, Betaine; 8, Methanol; 9, Glucose; 10, cAMP; 11, Tyrosine; 12, NAD^+^; 13, Niacinamide.

In the score plots of OPLS-DA analysis of myocardial NMR data (Fig. 9), good separations were achieved for each couple of NC and specific VB group. Color-coded loading plots (Fig. 9) and fold change plots (Fig. 8) revealed the increase of lactate, glucose, cAMP, and the decrease of creatine, lysine and taurine in the myocardial extracts due to VB treatment. The loading plots also revealed increased choline and betaine in middle and high dose VB groups at 2 hr. These metabolites were also severely changed according to univariate analysis as visualized by the fold change plots (Fig. 8f-j) and color table (S2 Fig.).

#### Metabolic changes in liver of VB treated rats

**Fig 10:**
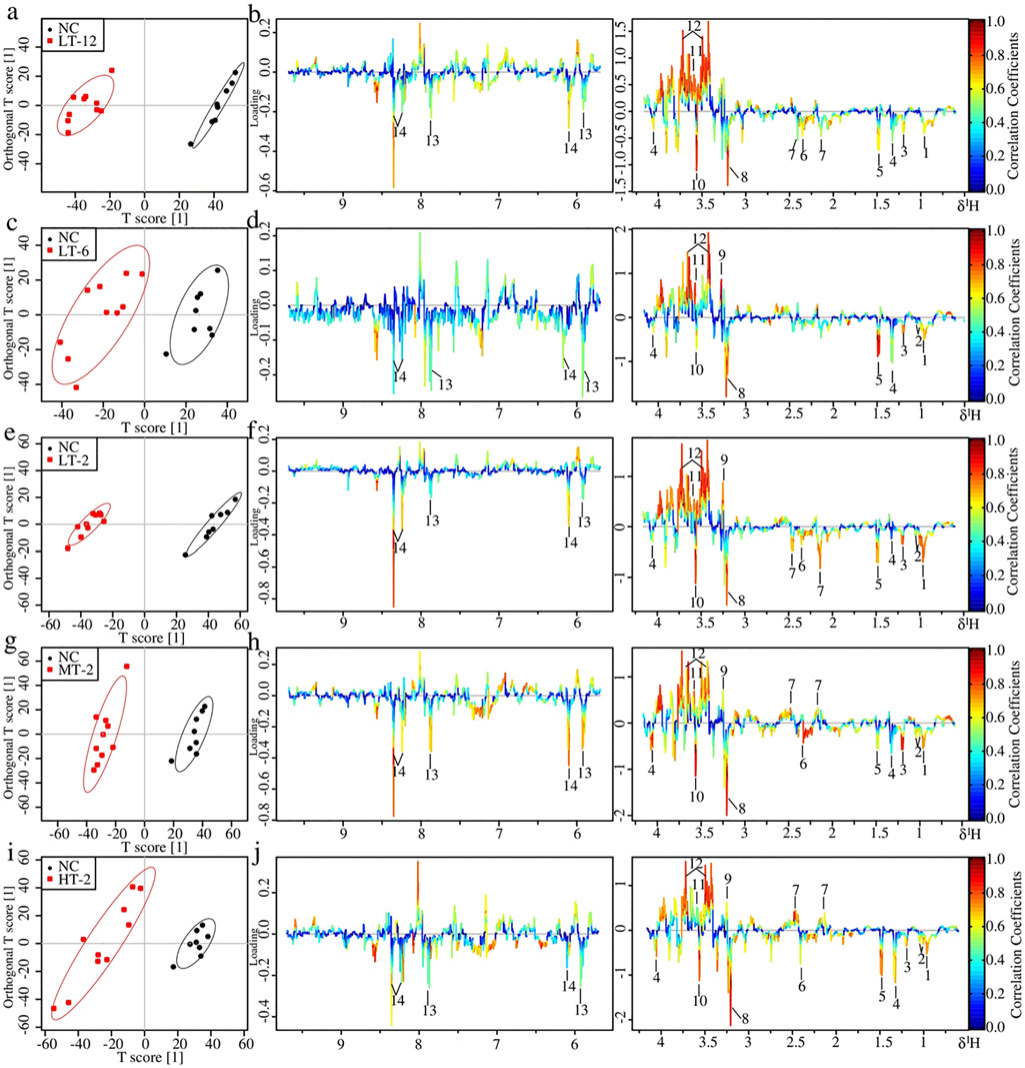
OPLS-DA analysis of liver extracts ^1^H NMR data of LT-12, LT-6, LT-2, MT-2 and HT-2 groups in couple with NC group: score plots (a, c, e, g and i) and loading plots (b, d, f, h and j). In score plots, ellipses around sample points stood for the 90% confidence. Metabolite variation was visualized by the loading plots, which are color-coded according to the absolute value of the correlation coefficient; a reddish signal indicates a more significant contribution to the class separation than a bluish signal. Metabolites: 1, Leu/Ile; 2, Valine; 3, 3-HB; 4, Lactate; 5, Alanine; 6, Succinate; 7, GSH; 8, Choline; 9, Betaine; 10, Glycine; 11, Glycerol; 12, Glucose; 13, Uridine; 14, Inosine.

Metabolic profiles of NC and specific VB group showed a clear separation in the score plots of OPLS-DA analysis of liver NMR data (Fig. 10). The loading plots (Fig. 10b, d, f, h and j) show the relevant changes in endogenous metabolites in the liver extracts that are responsible for the separation between the control group and the VB treated groups, including increased Leu/Ile, valine, 3-HB, alanine, succinate, choline and glycine, and decreased GSH, betaine, glycerol and glucose. These important differential metabolites were further tested and found to be mostly significant as visualized in the fold change plots (Fig. 8k-o) and color-table with associated *p*-values adjusted by Benjamini-Hochberg methods (S2 Fig.).

^1^H NMR revealed a number of alterations that have taken place in liver and concerned a large number of metabolites induced by VB, but no obviously time and dose dependently. It is likely to be affected by drug absorption and distribution. A further study on time and dose dependence hepatotoxicity of VB needs to be carried out.

Overall, low doses of VB produced no noticeable behavioral abnormalities but moderate metabolic disturbances in serum and myocardial extracts, and obviously metabolic disturbances in liver extracts of VB-administered rats: rats treated with low doses of VB were partial separated from other groups by OPLS-DA score plots (Fig. 6), and significant differences in compounds were observed in the corresponding loading plots. Middle and high doses of VB induced evident behavioral abnormalities and apparent metabolic disturbances in serum, myocardial extracts and liver extracts.

## Discussion

In this study, a ^1^H NMR-based metabolomics approach combined with behavioral observation, traditional clinical chemistry, electrocardiographical recordings, and histopathological evaluation were used to examine the perturbations induced by VB. In histological examination, abundant eosinophilic cytoplasm, marked inflammatory cell infiltrations and obviously dilated intercellular spaces were displayed in myocardium of VB-treated groups, along with increased levels of serum CK, MDA and cardiac ROS level. Hepatic histopathology showed moderate piecemeal necrosis and slight inflammatory cell infiltrations in the portal tracts in VB administered rats, which was evidenced by increased level of γ-GT in serum chemistry. OPLS-DA analyses of ^1^H NMR metabolomic profiles of serum, myocardial extracts and liver extracts revealed potential biomarkers and the affected metabolic pathways induced by VB: disturbance in energy metabolism, oxidative stress, mitochondrial dysfunction, and membrane damage.

### VB induced myocardial oxidative stress

The observed increase in MDA level in serum, in addition to the observed increase in taurine, ROS levels in myocardium from the VB treated group, suggested the presence of oxidation stress, which has been associated with mitochondrial dysfunction.

The significantly elevated ROS levels in myocardium demonstrated the oxidative stress occurred induced by VB treatment. Mitochondria are the primary sites of ROS, which make them especially vulnerable to oxidative damage that may affect mitochondrial membrane permeability, results in inner membrane permeabilization, outer membrane rupture, and cell apoptosis [33]. MDA, lipid peroxidation product, is an index of lipid peroxidation. The experimental low dosage VB treated rats showed significant elevation in MDA levels at 6 h and 12 h in serum. The increased levels of ROS and MDA directly demonstrated ROS generation and oxidative stress occur in the VB treated rats. An important scavenger of ROS *in vivo*, taurine exhibited decreased levels in VB treated rats, possibly due to its activities to counteract ROS, a primary mechanism in the initiation and progression of chronic heart failure and heart dysfunction [34]. Excessive generation of ROS by the respiratory chain [35] consumes a large amount of taurine.

The decreased levels of lysine observed in the myocardial extracts of rats given VB indicated the occurrence of oxidation stress after VB dosage. The lysine in albumin- and apo**B**100- containing particles can generate glycosylation end products or be further oxidized [36]. The observed decrease in serum free lysine levels might indicate oxidative stress injury in the myocardia of the rats administered VB.

### VB induced energy metabolism disturbance

Mitochondria are critical for energy supply through the oxidation of FFA and pyruvate and the conversion of chemical energy stored in acetyl-CoA into energy stored in adenosine triphosphate (ATP). ROS are capable of directly altering the permeability of the mitochondrial membrane, leading to mitochondrial dysfunction and a severe energy shortage.

Cardiac energy metabolism involves three consecutive steps: substrate utilization (fuel use, e.g., free fatty acids and glucose from food), oxidative phosphorylation (energy production by the mitochondrial respiratory chain), and ATP transfer and utilization by an energy-transfer mechanism termed the creatine kinase energy shuttle [37].

Sufficient substrate utilization is a prerequisite for normal cardiac function, as energy is required by the heart, and the heart’s primary energy supply is the ATP generated in mitochondria. The production of ATP involves contributions from fatty acids (60% to 90%) and glucose (10% to 40%). Increased levels of FFA indicate inhibition of fatty acid β-oxidation, resulting in an insufficient supply of acetyl-CoA to participate in the tricarboxylic acid (TCA) cycle, which necessitates the conversion of ketone bodies to acetyl-CoA to replenish acetyl-CoA. As a result, ketone bodies such as 3-HB, were markedly decreased in the sera of rats administered VB. The inhibition of fatty acid β-oxidation was also indicated by the observed decrease in the myocardial levels of lysine in VB-treated rats. As a strict ketogenic amino acid, lysine is required for the synthesis of L-carnitine, which is the only transporter of fatty acids to mitochondria [38].

Notably increased glucose, lactate and slightly increased alanine levels in myocardial extracts, and significantly increased glucose and decreased lactate, alanine levels in serum demonstrated an energy disturbance caused by the VB.

The significantly increased glucose in serum might due to increased rate of gluconeogenesis from alanine and lactate which were decreased in serum. Glucose gets into the myocardial cell to produce energy. But the increased glucose, lactate and alanine levels in the myocardial extracts demonstrated damage to the glucose aerobic oxidation pathways that normally provides most energy for myocardium through TCA cycle in mitochondria. In case of the hampered energy supply through this way, another means come to the rescue: glucose produces energy via anaerobic glycolysis with the production of lactate and alanine. The increased lactate and alanine levels in the myocardial extracts demonstrated an enhanced anaerobic glycolysis. The lactate and alanine levels were also increased in liver, which together with the decreased glucose level further demonstrated a facilitated anaerobic glycolysis and suggested an increased rate of glycogenolysis [39,40].

Decreased levels of creatine were observed in myocardial extracts from rats administered high doses of VB, consistent with levels observed in the early stages of heart failure [37]. The creatine-Pcr system is crucial for balancing the energy supply. When the energy demand outstrips its supply, CK catalyzes the transfer of the high-energy phosphate bond in phosphocreatine to adenosine diphosphate (ADP) to form ATP. As a result, phosphocreatine levels fall in order to maintain a normal level of ATP, but at the cost of an increased level of ADP. A high level of free ADP has been associated with cardiac dysfunction, as it inhibits the function of many intracellular enzymes [37].

The depletion of myocardial energy is the primary cause of chronic heart failure. In summary, myocardial energy metabolism was found to be impaired in rats administered VB. The myocardium consumes more energy than any other skeletal muscle. Because it is rich in mitochondria, the myocardium can directly utilize glucose, free fatty acids, and ketones to produce ATP. VB-induced ROS could impair mitochondrial function, leading to an insufficient energy supply. Without large glycogen and Pcr reserves, it is difficult for the myocardium to survive an energy crisis, resulting in cardiac dysfunction.

### VB induced hepatotoxicity

Histopathological inspection revealed VB induced moderate piecemeal necrosis and slight inflammatory cell infiltrations in the portal tracts of liver. OPLS-DA analyses of ^1^H NMR metabolomic profiles of liver extracts suggested that VB induced a great metabolic change concerning disturbance in protein synthesis and membrane damage.

Increased levels of amino acids (valine, Leu/Ile, glycine) were observed in liver of VB dosed rats, which could be ascribed to necrosis of liver parenchyma or to muscle proteolysis. The levels of choline were obviously increased in liver of VB treated groups. As a constituent of cell membranes and lipoprotein phospholipids, choline plays an important role in the integrity of cell membranes and lipid metabolism. In addition to be critical components of cellular membranes, phospholipids interact with all the membrane proteins and many non-membrane proteins, and mediate signal transduction. The increased levels of choline in VB treated rats suggested membrane damage, possibly leading to enhanced membrane permeability and altered membrane structure.

### Summary

**Fig 11:**
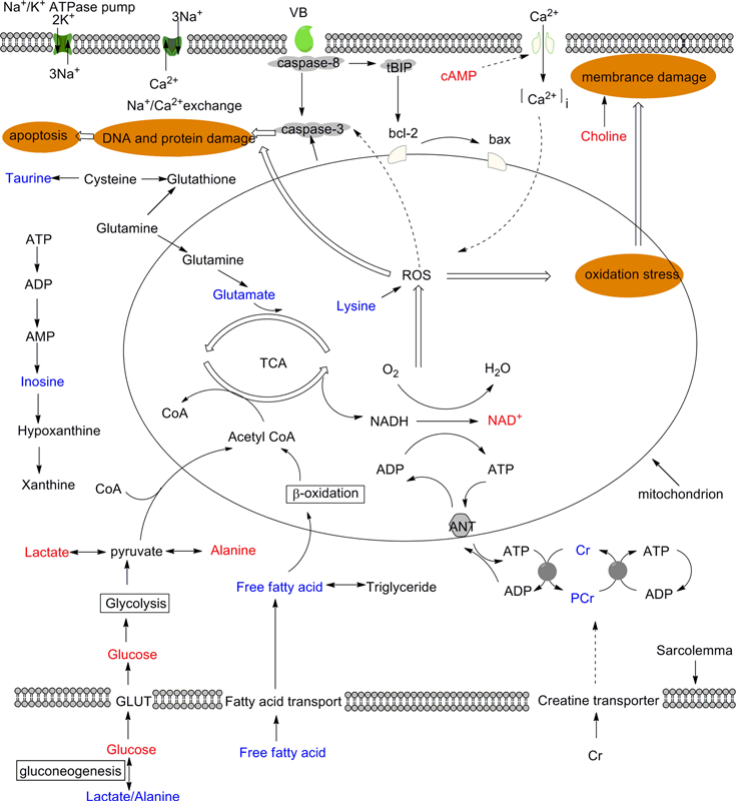
Schematic diagram of the metabolic pathways disturbed by VB. Metabolites in red and blue denoted the increase and decrease in their levels; those in black were not detected but are relevant.

Clinical chemistry, electrocardiographical recordings, histopathological evaluation, and metabolomics data demonstrated that oxidative stress, energy metabolism perturbation, mitochondrial dysfunction and membrane damage are involved in VB-induced cardiac damage (Fig. 11).

The primary active and toxic components of VB are the bufodienolides, which are cardioactive steroids that are structurally similar to digoxin. VB may induce cardiac acute toxicity by inhibiting the Na^+^/K^+^-ATPase pump and activating the mitochondrial apoptotic pathway, accompanied by excessive levels of ROS, can disrupt mitochondrial function and induce apoptosis [41].

## Conclusion

In this study, the toxic effects of VB were investigated for the first time using a ^1^H NMR-based metabolomics approach complemented by behavioral observation, clinical chemistry, electrocardiographical recordings, and histopathological evaluation. Metabolomic profiles of serum, myocardial extracts and liver extracts were analyzed using univariate and multivariate analysis statistical analysis. VB-induced metabolic patterns were found to significantly and dose-dependently deviate from normality. The behavioral abnormality and cardiac disturbance of VB peaked 2 hr after dosing and then attenuated gradually, demonstrating the self-healing ability of the body. OPLS-DA analysis identified several endogenous metabolites in serum, myocardial extracts and liver extracts that are closely related to VB-induced cardiac toxicity. Thus, oxidative stress, energy metabolism perturbation, mitochondrial dysfunction and membrane damage perturbation are involved in the toxic effects of VB. NMR-based metabolomics techniques allowed the characterization of metabolic events dynamically and holistically, demonstrating the great potential of these techniques in toxicity assessments and mechanistic studies.

## Supporting Information

S1 Fig 1D STOCSY analysis of myocardial extraction used to identify peaks of cAMP at 6.14 ppm. The degree of correlation across the spectrum has been color coded and projected on the spectrum. There is obviously covariance with peak at 6.14 ppm and 8.25 ppm.
(TIF)

S2 Fig Metabolites alterations in VB groups at different time relative to NC group for serum, hearts and livers. The unit cell was filled with color according to its logarithm of fold change value with significance for the variation denoted using “*”, “**”, “***” for *P*<0.05, *P*<0.01 and *P*<0.001. Metabolite abbreviations: LDL/VLDL: low-density-lipoproteins/very-low-density lipoproteins; Leu/Ile: Leucine/Isoleucine; 3-HB: 3-Hydroxybutyrate; NAGP: N-Acetyl Glycoproteins; Glu: glutamate; Gln: glutamine; GSH: glutathione; Phe: phenylalanine; Hyp: hypoxanthine; Xan: xanthine.
(TIF)

S1 File Zip archive of Bruker format ^1^H NMR data of rat serum sample spectra. (ZIP)

S2 File Zip archive of Bruker format ^1^H NMR data of rat heart sample spectra. (ZIP)

S3 File Zip archive of Bruker format ^1^H NMR data of rat liver sample spectra. (ZIP)

S4 File Zip archive of Bruker format HSQC data of serum, heart and liver. (ZIP)

S1 Table Assignment of the identified serum metabolites. (XLSX)

S2 Table Assignment of the metabolites identified in myocardial extracts. (XLSX)

S3 Table Assignment of the metabolites identified in liver extracts. (XLSX)

S4 Table The ARRIVE Guidelines Checklist. We followed the ARRIVE (Animal Research: Reporting of In Vivo Experiments) guidelines and the ARRIVE Checklist is available.
(PDF)
