# Supplementary figures and images for: Study of the Cardiotoxicity of Venenum Bufonis in Rats using an 1H NMR-Based Metabolomics Approach
Source: PLoS One. 2015 Mar 17;10(3):e0119515. doi: 10.1371/journal.pone.0119515 (PMC4363591; doi:10.1371/journal.pone.0119515)

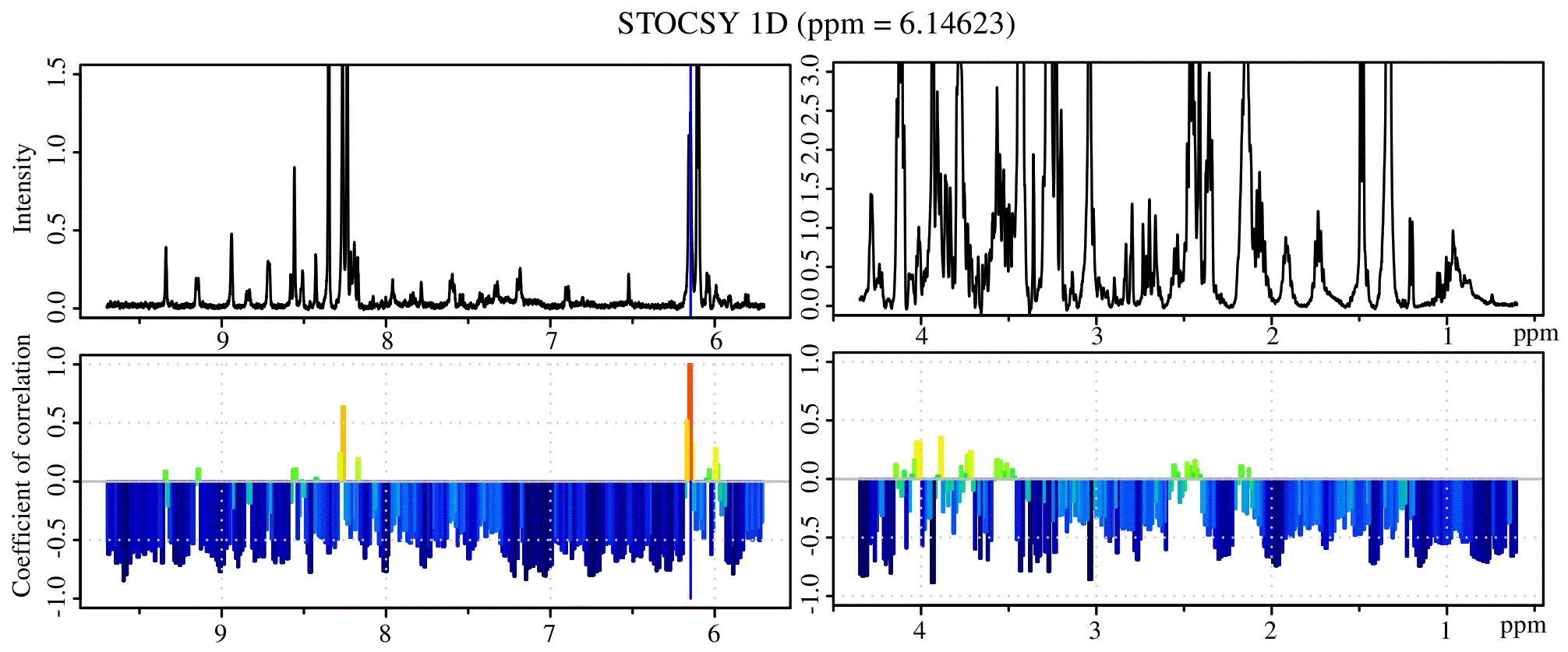

Supplement: S1 Fig — The degree of correlation across the spectrum has been color coded and projected on the spectrum. There is obviously covariance with peak at 6.14 ppm and 8.25 ppm. (TIF) [file pone.0119515.s001.tif]

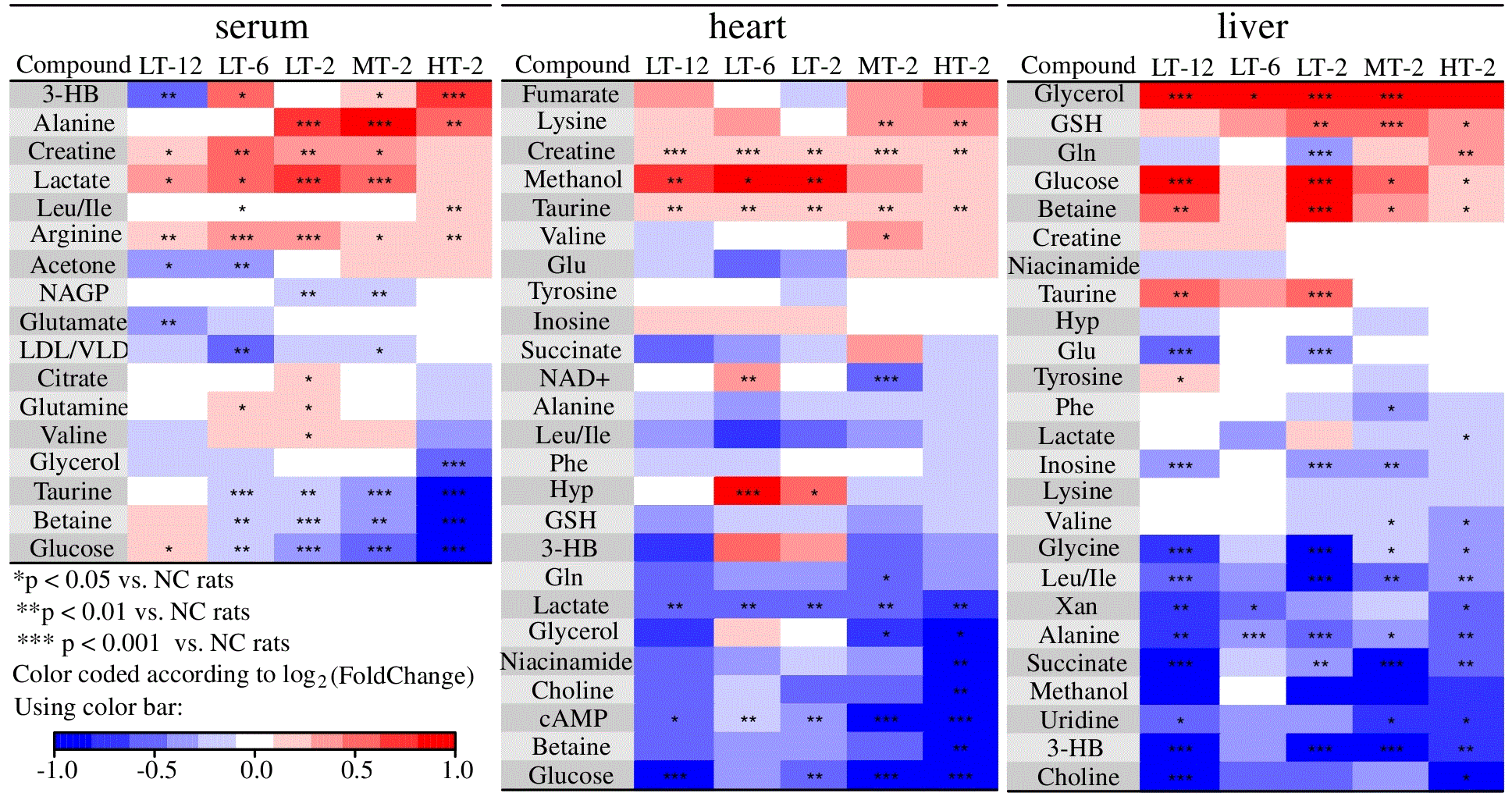

Supplement: S2 Fig — The unit cell was filled with color according to its logarithm of fold change value with significance for the variation denoted using “*”, “**”, “***” for P<0.05, P<0.01 and P<0.001. Metabolite abbreviations: LDL/VLDL: low-density-lipoproteins/very-low-density lipoproteins; Leu/Ile: Leucine/Isoleucine; 3-HB: 3-Hydroxybutyrate; NAGP: N-Acetyl Glycoproteins; Glu: glutamate; Gln: glutamine; GSH: glutathione; Phe: phenylalanine; Hyp: hypoxanthine; Xan: xanthine. (TIF) [file pone.0119515.s002.tif]
